# Rapid movement and instability of an invasive hybrid swarm

**DOI:** 10.1111/eva.12371

**Published:** 2016-04-27

**Authors:** Gregory J. Glotzbecker, David M. Walters, Michael J. Blum

**Affiliations:** ^1^ Department of Ecology and Evolutionary BiologyTulane UniversityNew OrleansLAUSA; ^2^ U.S. Geological SurveyFort Collins Science CenterFort CollinsCOUSA; ^3^ Tulane – Xavier Center for Bioenvironmental ResearchTulane UniversityNew OrleansLAUSA

**Keywords:** biological invasion, *Cyprinella*, introgression, moving hybrid zone, red shiner, species collapse

## Abstract

Unstable hybrid swarms that arise following the introduction of non‐native species can overwhelm native congeners, yet the stability of invasive hybrid swarms has not been well documented over time. Here, we examine genetic variation and clinal stability across a recently formed hybrid swarm involving native blacktail shiner (*Cyprinella venusta*) and non‐native red shiner (*C. lutrensis*) in the Upper Coosa River basin, which is widely considered to be a global hot spot of aquatic biodiversity. Examination of phenotypic, multilocus genotypic, and mitochondrial haplotype variability between 2005 and 2011 revealed that the proportion of hybrids has increased over time, with more than a third of all sampled individuals exhibiting admixture in the final year of sampling. Comparisons of clines over time indicated that the hybrid swarm has been rapidly progressing upstream, but at a declining and slower pace than rates estimated from historical collection records. Clinal comparisons also showed that the hybrid swarm has been expanding and contracting over time. Additionally, we documented the presence of red shiner and hybrids farther downstream than prior studies have detected, which suggests that congeners in the Coosa River basin, including all remaining populations of the threatened blue shiner (*Cyprinella caerulea)*, are at greater risk than previously thought.

## Introduction

Hybrid zones are areas of contact between two genetically distinct populations where hybridization occurs (Allendorf et al. 2001). In certain cases, a hybrid swarm may develop within a hybrid zone, with populations that consist predominantly of hybrids, arising from backcrossing with parental types and mating among hybrids (Seehausen 2006). Hybrid swarms can arise and become highly unstable as a result of disruptive shifts in environmental conditions or ecological interactions. Incomplete prezygotic (e.g., weak assortative mating) and postzygotic isolation (e.g., little or no selection against hybrids) can erode steep coincident clinal transitions between parental entities to produce unimodal phenotypic and genotypic distributions (Endler 1977; Harrison 1990; Barton and Gale 1993; Arnold 1997; Harrison and Bogdanowicz 1997; Jiggins and Mallet 2000). Other factors like elevated hybrid fitness and the rise of advantageous traits in admixed populations can accelerate genetic homogenization (Arnold 1997; Barton 2001; Coyne and Orr 2004; Bettles et al. 2005; Hall et al. 2006). Consequently, changes in abiotic and biotic conditions that weaken reproductive isolation or that favor hybrids can trigger the formation, movement, and expansion of hybrid swarms. Species collapse in African rift lake cichlids and European whitefish, for example, was precipitated by eutrophication relaxing sexual selection among co‐occurring species (Seehausen et al. 1997, 2008; Seehausen 2006; Bittner et al. 2010; Vonlanthen et al. 2012). The introduction of a non‐native crayfish capable of disrupting premating barriers, such as nesting preferences, is thought to have precipitated the collapse of sympatric lentic and benthic threespine stickleback into a hybrid swarm (Taylor et al. 2006). Anthropogenic habitat modification has also led to the formation of hybrid swarms, as observed between native westslope cutthroat trout (*Oncorhynchus clarkii lewisi*) and introduced rainbow trout (*O. mykiss*) (Yau and Taylor 2013), and naturally sympatric populations of alewife (*Alosa pseudoharengus*) and blueback herring (*A. aestivalis*) following the construction of a dam on the Roanoke River (North Carolina, Virginia, USA; Hasselman et al. 2014). Similarly, deforestation and related shifts in competitive interactions appear to have promoted the movement and modification of hybrid swarms involving warning color races of *Heliconius erato* butterflies (Blum 2002, 2008).

Disruptive shifts that result in unstable hybrid swarms can lead to the rapid loss of biodiversity. Unstable hybrid swarms that arise following the introduction of non‐native species are of particular concern because native congeners can be quickly overcome (Rhymer and Simberloff 1996; Huxel 1999; Mooney and Cleland 2001; Epifanio and Philipp 2000; Wolf et al. 2001; Hall et al. 2006; Coleman et al. 2014). A loss of reproductive isolation between morph pairs of European whitefish (*Coregonus lavaretus* (L.)), for example, could occur within three generations following an invasion event (Bhat et al. 2014). Hybridization with the imperiled Pecos pupfish (*Cyprinodon pecosensis*) following the introduction of sheepshead minnow (*Cyprinodon variegatus*) to the Pecos River drainage (Texas and New Mexico, USA) resulted in the spread of hybrids across more than half of the range of the Pecos pupfish in less than five years (Echelle and Connor 1989; Childs et al. 1996). Within a decade of introduction, hybridization with the invasive crayfish *Orconectes rusticus* led to the displacement of native *O. propinquus* in lakes across Wisconsin (USA; Perry et al. 2002). Hybrid *Spartina* cordgrasses have also rapidly overtaken native congeners in San Francisco Bay (California, USA) and elsewhere in the world (Ayres et al. 2008a,b; Castillo et al. 2010; Strong and Ayres 2013).

Understanding the movement and stability of hybrid swarms that have formed following the introduction of non‐native species can guide strategies to prevent the loss of biodiversity (Phillips 2015). Like patterns of spatial variation in observable traits, patterns of temporal variation reflect the influence of genetic and ecological factors on the integrity of parental boundaries (Barton 1983; Barton and Hewitt 1985, 1989; Harrison 1990; Barton and Gale 1993; Arnold 1997). Patterns of temporal variation also offer perspective on rates of genetic homogenization and risk of displacement (Ellstrand and Schierenbeck 2000; Epifanio and Philipp 2000; Sakai et al. 2001; Wolf et al. 2001; Perry et al. 2002; Hall et al. 2006). In this study, we examined the movement and stability of a recently formed hybrid swarm (Walters et al. 2008) involving native blacktail shiner (*Cyprinella venusta stigmatura*) and introduced non‐native red shiner (*Cyprinella lutrensis*) in the Upper Coosa River basin (Alabama, Georgia, Tennessee, USA). Estimates of movement and descriptions of stability of the swarm over time have largely been inferred from historical collection records (Walters et al. 2008; Blum et al. 2010; Ward et al. 2012). Evidence of clinal discordance in the distribution of multilocus microsatellite genotypes, mtDNA haplotypes, and phenotypic traits suggests, however, that historical records do not fully capture patterns of instability or the extent and rate of movement (Ward et al. 2012). Genotypic and phenotypic discordance indicates that retention of parental phenotypes is likely masking the full extent of hybridization in the system (Ward et al. 2012). Here, we compare clinal variation in nuclear and mitochondrial genetic markers, as well as phenotypic traits, over six years to better understand the progression of the *C. lutrensis x C. venusta* hybrid swarm in the Coosa River. Doing so enabled us to infer the tempo and pace of change according to observable variation and cryptic introgression, which in turn enabled us to better assess the risk that the invasion poses to vulnerable populations of native congeners.

## Materials and methods

### Study system and collections

**Figure 1:**
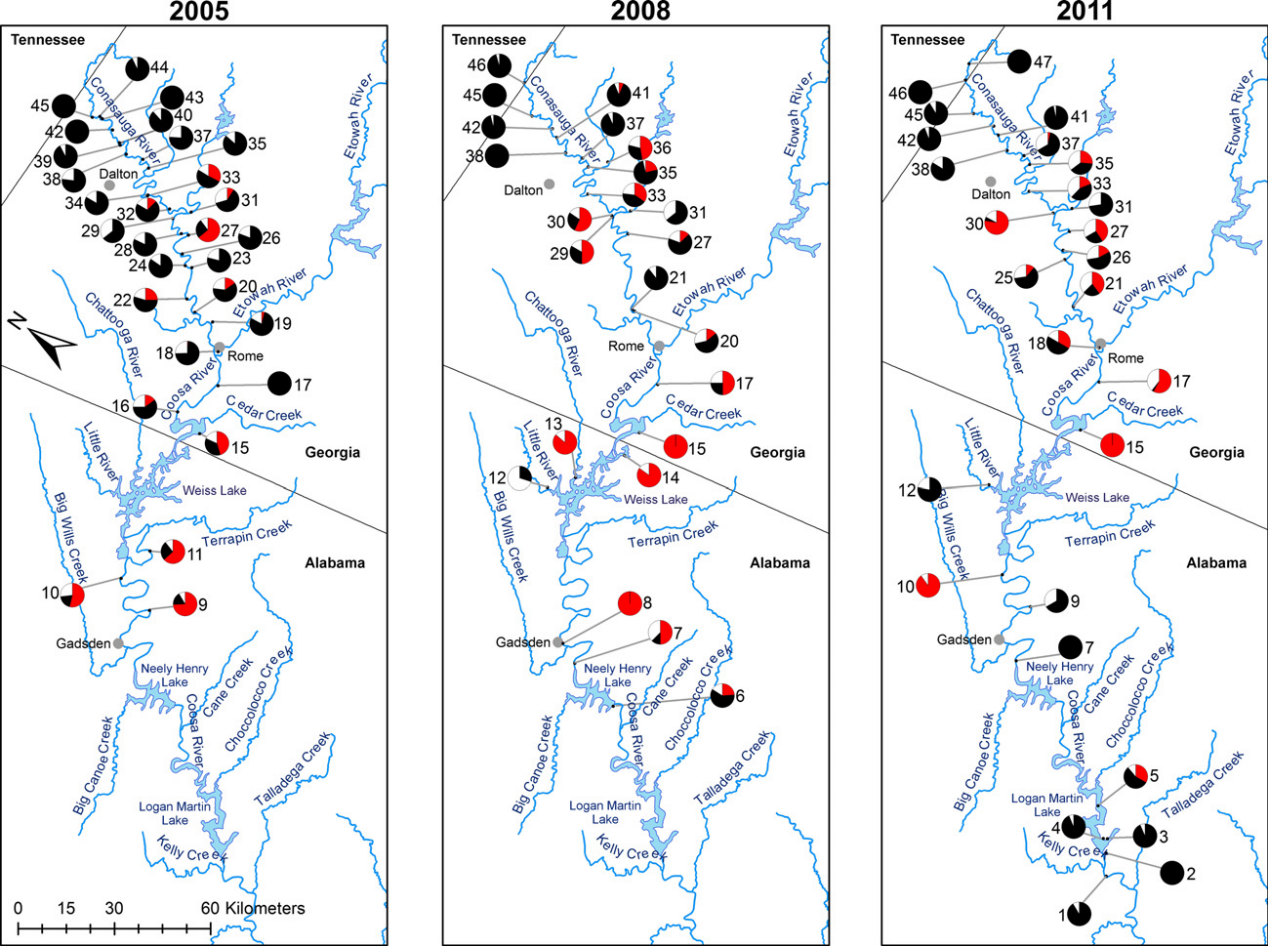
Forty‐seven locations sampled between 2005 and 2011, along a 477‐km transect of the upper Coosa River basin including the Coosa River, Oostanaula River, and Conasauga River (Alabama, Georgia, Tennessee; USA). Relative proportions of multilocus genotypes recovered at each collection site are provided for *C. lutrensis* (red), *C. venusta* (black), and *C. lutrensis* x *C. venusta* hybrids (white).

Red shiners were first recorded in the upper Coosa River basin at a site in Weiss Lake (Alabama, USA; Fig. 1) in 1974 (Walters et al. 2008). Annual surveys first documented hybridization with blacktail shiner in the mainstem Coosa River in 1998 (Burkhead and Huge 2002; Walters et al. 2008) and indicate that the leading edge of a resulting hybrid swarm has progressed upstream at rates of up to 31 km per year (Walters et al. 2008). Ward et al. (2012) subsequently found evidence of phenotype–genotype discordance indicating that parental phenotypes are often retained in admixed individuals and that introgression extends beyond the observable upstream edge of the hybrid swarm.

Following collection records and prior genetic studies (Walters et al. 2008; Ward et al. 2012), we obtained 1324 *Cyprinella* in the summer months of 2008 and 2011 to build on sampling conducted in 2005 (Ward et al. 2012) that yielded 1078 specimens (Table 1). Cumulatively, from 2005 through 2011, sampling locations included a total of 47 sites over a 477‐km transect spanning sites south of Logan Martin Lake in northern Alabama to sites on the Conasauga River north of the Georgia‐Tennessee state line (Fig. 1). We extended the transect progressively farther south over time, however, to capture the downstream extent of the hybrid swarm in the system. For example, in 2008 the southern terminus of the transect was 100 km south of Weiss Lake at Neely Henry Dam, whereas in 2011 we sampled 100 km farther south at Logan Martin Lake because preliminary analyses showed that hybrids were present in Neely Henry Lake in 2008. River distances between collection sites were measured from satellite imagery using Google Earth v7.1.5.1557 (Google Inc., Mountain View, CA, USA). At each site, fish were collected by seine netting and then immediately placed in 95% ethanol for morphological and genetic analysis.

**Table 1:** Summary data for 47 locations where *Cyprinella* were sampled in the Upper Coosa River Basin. Population numbers correspond with the numbers on Fig. 1. Population 8 and Population 15 correspond to the East Gadsden Boat Ramp and Brushy Branch (Weiss Lake) collection sites, respectively

Population	Latitude	Longitude	2005				2008				2011			
			*n*	Phenotype	mtDNA	Msat	*n*	Phenotype	mtDNA	Msat	*n*	Phenotype	mtDNA	Msat
	(N)	(W)		(*n*)	(*n*)	(*n*)		(*n*)	(*n*)	(*n*)		(*n*)	(*n*)	(*n*)
1	33.390	−86.378									23	23	22	23
2	33.427	−86.325									11	11	11	11
3	33.447	−86.290									14	14	8	14
4	33.457	−86.296									18	18	14	16
5	33.522	−86.229									9	9	9	9
6	33.786	−86.065					30	30	29	25				
7	33.943	−86.028					42	42	38	40	7	7	5	7
8	34.002	−86.002					1	1	0	1				
9	33.997	−85.881	35	15	35	35					3	3	3	3
10	34.113	−85.853	179	36	176	177					10	2	9	10
11	34.091	−85.744	26	17	26	26								
12	34.288	−85.669					12	12	10	10	35	8	34	35
13	34.239	−85.601					29	29	23	29				
14	34.162	−85.472					33	33	28	33				
15	34.165	−85.396	15	10	11	11	1	1	1	1	2	1	2	2
16	34.251	−85.381	5	5	5	5								
17	34.200	−85.256	20	19	20	20	16	16	15	16	46	44	37	37
18	34.255	−85.178	110	7	106	106					13	13	13	12
19	34.315	−85.118	129	123	129	128								
20	34.371	−85.125	26	23	26	26	29	29	21	28				
21	34.380	−85.124					19	19	16	19	108	63	94	87
22	34.411	−85.107	68	51	64	64								
23	34.450	−85.027	19	12	19	19								
24	34.468	−85.033	26	22	26	26								
25	34.476	−85.030									44	41	43	43
26	34.494	−85.011	26	26	26	26					31	31	27	28
27	34.510	−84.958	39	34	39	39	32	32	29	31	26	16	24	26
28	34.529	−84.966	29	25	28	28								
29	34.573	−84.945	45	36	45	44	7	7	7	6				
30	34.577	−84.942					58	58	47	58	122	108	112	115
31	34.541	−84.901	14	14	14	14	14	14	12	14	11	11	11	11
32	34.595	−84.928	24	21	24	24								
33	34.667	−84.931	43	37	43	43	27	27	27	26	37	33	31	36
34	34.667	−84.933	12	8	12	12								
35	34.709	−84.868	22	0	22	22	28	28	28	28	34	34	30	33
36	34.672	−84.825					19	19	16	19				
37	34.736	−84.857	26	25	25	25	54	54	49	44	29	26	22	29
38	34.783	−84.872	39	23	39	39	25	25	25	25	36	36	33	36
39	34.811	−84.861	26	26	26	26								
40	34.817	−84.857	17	6	17	17								
41	34.828	−84.851					16	16	14	16	29	28	29	29
42	34.853	−84.838	22	18	22	22	25	25	25	25	32	31	30	32
43	34.895	−84.829	11	8	11	11								
44	34.904	−84.828	14	14	14	14								
45	34.920	−84.842	11	9	10	10	1	1	1	1	22	22	14	22
46	34.992	−84.778					24	24	24	22	23	23	23	23
47	35.010	−84.734									7	7	7	7
Total			1078	670	1060	1059	542	542	485	517	782	663	697	736

### Phenotypic trait measurements

We measured four phenotypic traits that identify and distinguish *C. lutrensis* and *C. venusta* (Boschung and Mayden 2004) following Ward et al. (2012). These traits include standard length (SL), maximum body depth (BD), lateral line scale count, and caudal spot intensity. Only individuals larger than 30 mm SL were measured due to the difficulty of obtaining accurate lateral line scale counts from smaller individuals. For the 2005 collections, site 37 was excluded from phenotypic analysis because all specimens were <30 mm in length. However, these fish were included in genetic analysis. Following Ward et al. (2012), subsequent analyses involved use of the ratio of SL to BD as a body size index, corresponding to the residuals for each specimen from the linear regression of BD on SL. And, because lateral line scale counts, size, and caudal spot intensity are highly correlated for both species (Ward et al. 2012), we conducted a principal component analysis (PCA) on the entire dataset of individuals from 2005, 2008, and 2011, to derive an overall phenotypic score for each individual. Trait decomposition yielded a single principal component that explained 81.6% of phenotypic variation.

### Microsatellite genotyping and mtDNA‐RFLP assays

We extracted DNA and amplified targeted regions of the nuclear and mitochondrial genomes following Walters et al. (2008). Briefly, for each individual, genomic DNA was extracted from approximately 0.05 g of preserved fin tissue using DNeasy kits (Qiagen, Valencia, CA, USA). Polymerase chain reaction (PCR) mixtures for amplification of both the complete *cytochrome b* gene (*cyt b*) and seven microsatellite loci (*Nme* 25C8.208, *Nme* 18C2.178, *Nme* 24B6.191, *Nme* 24B6.211, Rhca20, Rhca24, Can6EPA) included 2.5 mm MgCl_2_, 2.5 mm of each dNTP, 0.5 units Taq DNA polymerase (Invitrogen, Carlsbad, CA, USA), 0.5 μm PCR buffer (Invitrogen), and 0.5 μm of either the oligonucleotide primers GLU and THR for *cyt b* (Schmidt et al. 1998) or one of seven microsatellite primer pairs (Dimsoski et al. 2000; Burridge and Gold 2003; Girard and Angers 2006; Walters et al. 2008). PCR annealing temperatures were adjusted according to Walters et al. (2008), and amplification of microsatellite loci involved use of fluorescently labeled forward primers. Microsatellite PCR products were characterized on an ABI 3730xl (Applied Biosystems Inc., Foster City, CA, USA) and scored with GeneMarker v1.90 software (Softgenetics, State College, PA, USA) against a LIZ 600 size standard (Applied Biosystems^®^, Waltham, MA, USA). The *cyt b* PCR product was restricted with *HinfI* (New England Biolabs, Ipswich, MA, USA) following Walters et al. (2008) to generate and score unique fragment size profiles for *C. venusta* and *C. lutrensis* that were electrophoretically screened on agarose gels. All individuals were assigned species‐level mtDNA ancestry from the restriction profiles of *cyt b* amplicons.

### Analysis of admixture and genetic differentiation from microsatellite variation

**Figure 2:**
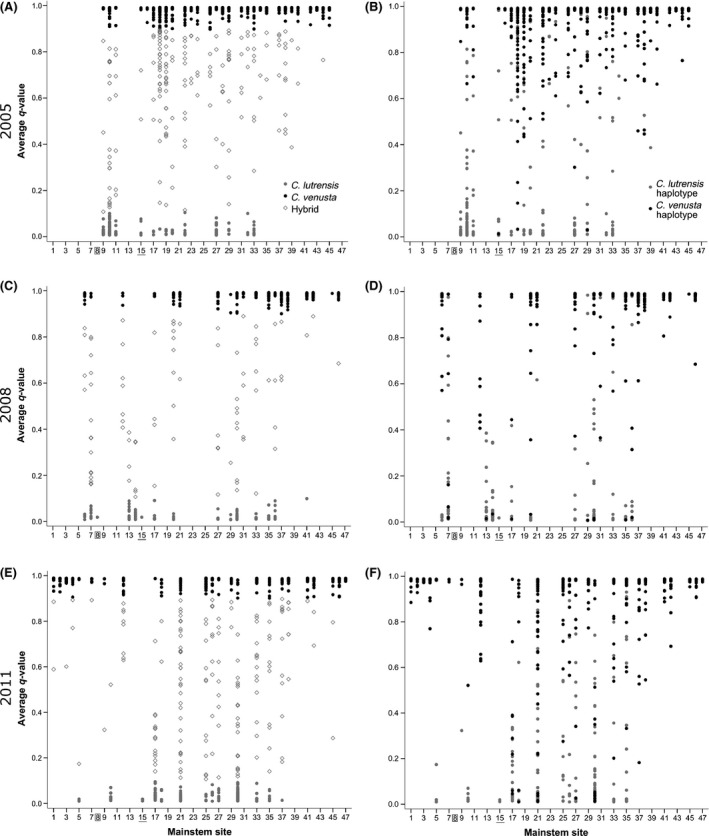
Comparison of multilocus genotype (average q‐value) against dominant phenotype (A), (C), (E) and mitochondrial haplotype (B), (D), (F) for *Cyprinella* sampled from 47 sites (as listed in Table 1) across the study transect in 2005, 2008, and 2011. Collection site #8 (East Gadsden Boat Ramp) represents the southern terminus of the 2005, 2008, and 2011 cline models, and collection site #15 (Brushy Branch) represents the northern terminus of the 2011 southern cline models.

Multilocus admixture profiles for all individuals were generated using the program Structure v2.3.4 (Pritchard et al. 2000). We undertook preliminary analyses to evaluate the relative contribution of individual loci to admixture profiles by comparing the results of runs generated using all seven loci with results of runs involving sequential removal of individual loci following Ward et al. (2012). No loci were found to bias the results, and all loci were informative. Five independent runs at *K* = 2 (i.e., representing the parental species) were subsequently executed to characterize admixture profiles for the 2005, 2008, and 2011 collections. For all runs, data were collected over 100 000 iterations, following a 50 000 iteration burn‐in, under an admixture model of co‐ancestry and correlated allele frequencies (Falush et al. 2003). Each run was parameterized following a model of admixture and correlated allele frequencies, and average assignment values to each genetic cluster were then calculated for all individuals. Following Walters et al. (2008) and Ward et al. (2012), individuals were then assigned to an admixture category according to average assignment values to the first cluster based on the following ranges of values: (i) red shiner, 0.90–1.0; (ii) blacktail shiner, 0–0.10; and (iii) hybrid, 0.11–0.89. For all individuals, multilocus genotype was plotted against dominant phenotype (i.e., red shiner, blacktail shiner, hybrid) and mtDNA haplotype to illustrate the nature and extent of hybridization in the study area (Fig. 2).

### Clinal analysis of phenotype and molecular data

We used the R package *HZAR* (Derryberry et al. 2014) to fit clines to admixture profiles based on multilocus microsatellite genotypes, the relative frequencies of mtDNA haplotype assignments, and dominant phenotype according to PCA scores. *HZAR* fits clines using a Metropolis–Hastings Markov chain Monte Carlo (MCMC) algorithm. Autofit functions allow for automated model selection from a set of nested cline models using Akaike's Information Criterion (AIC; Akaike 1973). To assess concordance and coincidence, we constructed maximum likelihood (ML) profiles for cline widths and cline centers, respectively. Estimates of cline center and width corresponding to the largest logLik values were selected and used to calculate AIC scores. For each sample year, AIC scores for cline center and width were then calculated for each of the three cline models using the equation: AIC = −2(logLik)+2*K*. Concordance and coincidence for individual clines for each sample year (e.g., the 2005 genotype, mtDNA, and phenotype clines) and across sample years (e.g., the 2005, 2008, and 2011 mtDNA clines) were then assessed for significance by comparing differences in AIC scores (ΔAIC). Following Burnham & Anderson (2002) and Anderson (2008), if the AIC score of one cline center differed by ≥2 compared to another cline center, then the clines were considered noncoincident. The same criteria were used for comparisons of cline widths. Additionally, 2 log likelihood intervals for cline center and width were calculated in *HZAR* for all sample years. Such intervals provide estimation of support for modeled cline centers and widths, similar to confidence and credibility intervals. Unless otherwise noted, for all analysis we examined northward (i.e., upstream) clines, excluding samples obtained from sites south of East Gadsden Boat Ramp (Population #8; Table 1) to avoid complications that can arise from cline fitting across multiple transitions. Thus, northward clinal variation across the hybrid swarm in 2005, 2008, and 2011 was modeled with East Gadsden Boat Ramp representing the southern terminus of a 352‐km transect for estimates of cline center and widths. For the 2011 dataset, we also fit southward downstream clines, excluding samples obtained from sites north of Brushy Branch (Population #15; Table 1). Therefore, the southward cline models for 2011 describe a 246‐km transect with Brushy Branch serving as the northward terminus.

## Results

### Hybridization across years

For all three collection years, we detected spatial structure in the relative frequencies of pure parental and hybrid multilocus admixture profiles based on assignment values (Figs 1 and 2). The proportion of individuals exhibiting admixed multilocus genotypes averaged 15% in 2005, 17% in 2008, and 22% in 2011. Estimates of genotypic admixture at individual collection sites ranged as high as 36% in 2005, 38% in 2008, and 39% in 2011 (Figs 1 and 2). Admixture proportions indicate that the majority of hybrids were later‐generation and backcrossed individuals, with a bias more frequently observed in the direction of *C. venusta* (Fig. 2).

The proportion of individuals exhibiting evidence of hybridization (i.e., genotypic admixture, nuclear–mitochondrial discordance, genotype–phenotype discordance) averaged 15% in 2005, 23% in 2008, and 36% in 2011. Estimates of hybridization at individual collection sites ranged as high as 36% in 2005, 70% in 2008, and 100% in 2011 (Figs 1 and 2). In 2005, the majority of hybrids exhibiting nuclear–mitochondrial discordance harbored a *C. lutrensis* mtDNA haplotype and *C. venusta* dominant genotype (Fig. 2). In 2008 and 2011, however, hybrids exhibiting nuclear–mitochondrial discordance harbored haplotypes and genotypes of both species at similar frequencies (Fig. 2). Hybrids exhibiting genotype–phenotype discordance most often exhibited a *C. lutrensis* dominant genetic profile and *C. venusta* phenotype in 2005 and 2011. In 2008, hybrids exhibiting genotype–phenotype discordance harbored phenotypes and genotypes of both species at similar frequencies (Fig. 2).

### Clinal variation across the Upper Coosa River basin

**Figure 3:**
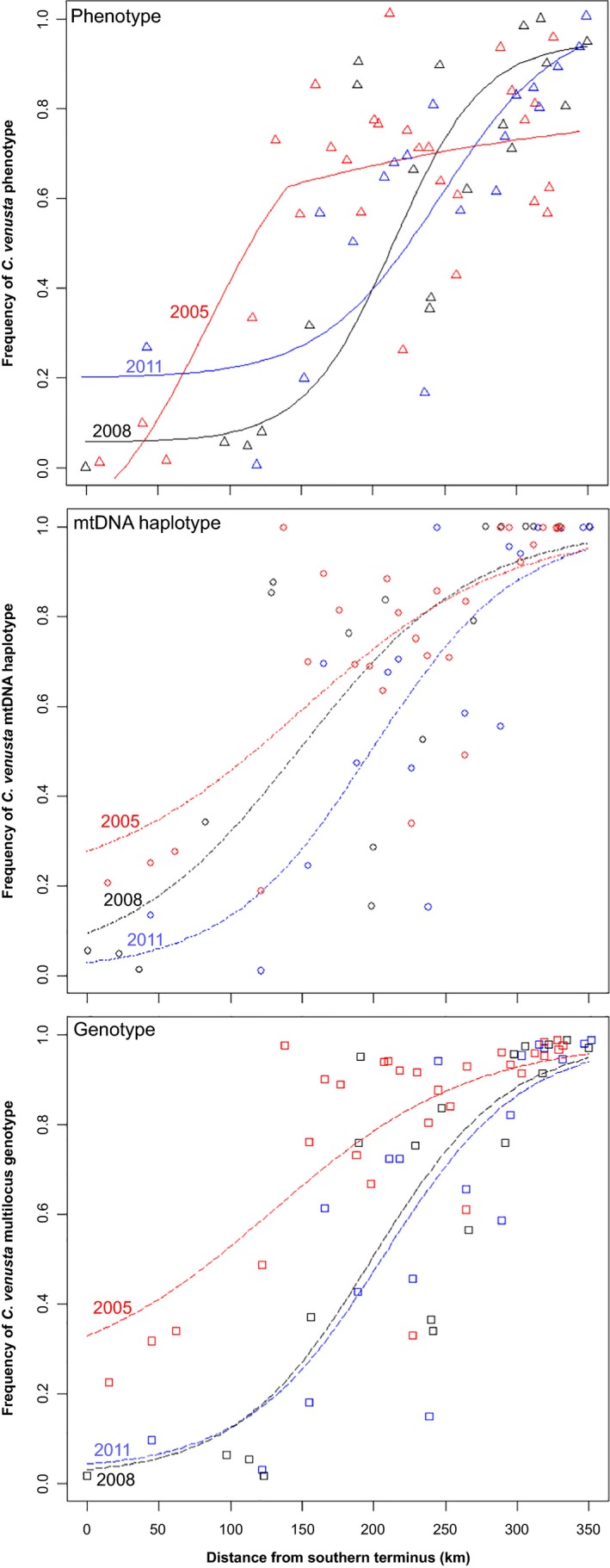
Clinal changes in frequencies of *Cyprinella* phenotype, mtDNA haplotype, and microsatellite multilocus genotype between 2005 and 2011. Top: phenotype cline models, Mid: mtDNA haplotype cline models, Bottom: multilocus genotype cline models. East Gadsden Boat Ramp was the southern terminus of the transect over which the clines were estimated.

Individuals exhibiting parental *C. lutrensis* phenotypes, haplotype, and genotypes were numerically dominant at the southern terminus (East Gadsden Boat Ramp) and decreased in frequency toward the northern terminus of the truncated upstream transect (Fig. 2). In all of the measured phenotypic traits, traits exhibited by individuals at the southern terminus (East Gadsden Boat Ramp, Population #8) were significantly different from those at the northern terminus (*P* = 0.000). All individuals exhibited a *C. venusta* haplotype at distances >300 km to the north of the southern terminus (Figs 2 and 3). No pure parental *C. lutrensis* genotypes were detected at distances upstream of 252 km from the southern terminus in 2005, 315 km in 2008, and 340 km in 2011 (Figs 2 and 3). However, hybrid genotypes were recovered at sites more than 60 km beyond the northernmost extent of parental *C. lutrensis* genotypes and phenotypes in 2005, and more than 30 km beyond the northernmost extent of parental *C. lutrensis* genotypes and phenotypes in 2008 and 2011 (Figs 2 and 3).

Cross‐year comparisons detected northward shifts in cline centers and cline widths increasing and decreasing over time (Table 2). Models estimated the center of the phenotypic cline to be 117 km from the southern terminus in 2005, 214 km in 2008, and 250 km in 2011 (Table 2, Fig. 3). Estimated widths of the phenotypic clines were 221 km in 2005, 121 km in 2008, and 168 km in 2011 (Table 2, Fig. 3). Models of haplotype variation estimated the cline center to be 156 km from the southern terminus in 2005, 202 km in 2008, and 197 km in 2011 (Fig. 3). Model estimates indicate that the width of the mtDNA cline decreased from 264 km in 2005 to 175 km in 2008, and then increased to 203 km in 2011 (Fig. 3, Table 2). The multilocus genotype cline center was estimated to be at 126 km in 2005, 197 km in 2008, and 208 km in 2011 (Table 2, Fig. 3). The estimated width of the multilocus cline declined from 280 km 2005 to 188 km 2008, after which it increased slightly to 192 km 2011 (Table 2).

**Table 2:** Comparison of genetic (mtDNA, Msat) and phenotypic (phenotype) clines models across the *C. lutrensis x C. venusta* hybrid swarm from 2005 to 2011. Number of model parameters (npar), Akaike's Information Criterion (AIC), center AIC score (AICc), width AIC score (AICw), 2 log likelihood interval center (2LLc), 2 log likelihood interval width (2LLw). All distance measures are expressed in kilometers. Cline centers are expressed as the fluvial distance from the southern terminus at East Gadsden Boat Ramp (Population #8), except for the 2011 South clines, which are expressed as the fluvial distance from the northern terminus at Brushy Branch (Population #15)

Cline model	Cline center	2LLc	Cline width	2LLw	npar	Center LL	Width LL	AICc	AICw
**2005**									
Phenotype	116.667	−29.824–128.222	220.833	102.840–414.970	11	−221.396	−224.634	464.792	471.269
mtDNA	156.250	23.357–228.709	264.375	105.048–409.913	2	−2.737	−2.737	9.475	9.475
Msat	126.250	21.191–205.859	280.000	126.152–414.969	2	−3.849	−3.849	11.698	11.698
**2008**									
Phenotype	213.542	207.858–218.770	121.250	106.714–142.710	3	−63.212	−63.194	132.424	132.388
mtDNA	202.083	99.096–258.598	175.000	83.532–409.961	2	−2.825	−2.825	9.649	9.649
Msat	197.917	138.254–238.974	187.500	101.905–414.140	2	−3.832	−3.831	11.663	11.662
**2011**									
Phenotype	250.000	244.141–255.649	167.500	148.364–195.265	3	−60.526	−60.522	127.052	127.044
mtDNA	197.368	78.537–259.116	202.632	89.814–414.941	2	−2.390	−2.388	8.779	8.775
Msat	207.895	138.091–248.686	192.105	100.226–414.981	2	−3.367	−3.367	10.733	10.733
**2011 South**									
Phenotype	89.737	79.523–93.018	57.105	9.747–57.996	3	78.989	78.986	−151.978	−151.973
mtDNA	116.667	−27.234–199.310	143.750	46.848–304.760	2	−1.098	−1.098	6.197	6.197
Msat	122.917	16.905–187.952	150.000	0.021–304.996	2	−1.809	−1.809	7.618	7.618

The cline models describing genotypic, phenotypic, and haplotype distributions along the Coosa mainstem transect exhibited both concordance and discordance across sample years (Fig. 3, Figure S1A–C). The mtDNA and multilocus genotype cline models did not statistically differ in either center or width (Table 2), whereas both consistently differed from the estimated phenotypic cline centers and widths (Table 2). Comparisons of clines over time also indicate that the centers of all three clines have shifted northward over time. However, the phenotypic clines exhibited a higher rate of northward advancement; in 2008, the center of the phenotypic cline advanced to the north of the mtDNA and multilocus cline centers (Table 2; Figure S1B). A similar pattern was detected in 2011, when the center of the phenotypic cline was estimated to be even farther upstream of the mtDNA and multilocus genotype cline centers (Table 2; Figure S1C). The width of the phenotypic cline remained narrower than the other clines in 2008 and 2011 (Table 2; Figure S1A–C).

### Southern extent of red shiner and hybrids in the Upper Coosa River basin

**Figure 4:**
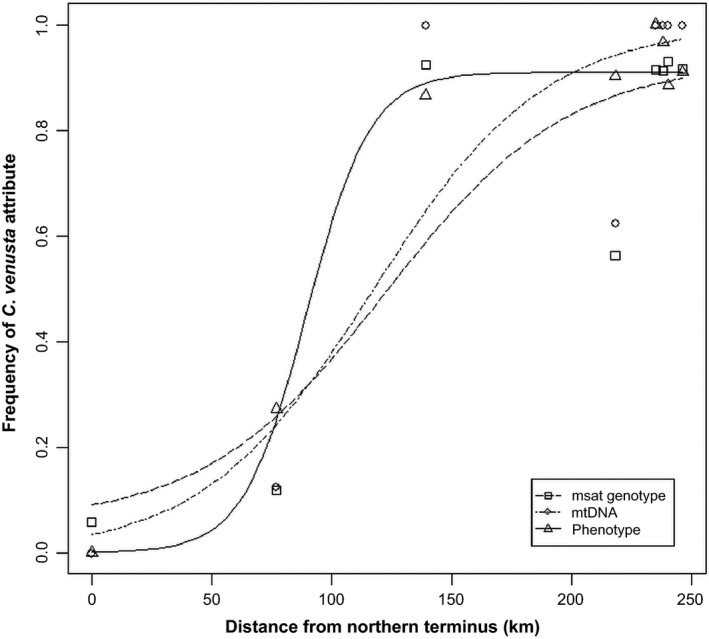
Southerly clinal changes in the frequencies of *Cyprinella* phenotype, mtDNA haplotype, and microsatellite multilocus genotype in 2011. Brushy branch was the northern terminus of the transect over which the clines were estimated.

In 2008 and 2011, we expanded our sampling to areas south of East Gadsden Boat Ramp to document the downstream distributional extent of red shiner and hybrids in the Upper Coosa River (Table 1, Fig. 1). In 2008, we detected red shiner and *C. lutrensis x C. venusta* hybrids at distances up to 39 km south of East Gadsden Boat Ramp (Figs 1 and 2). The prevalence of red shiner and hybrids varied from site to site downstream of East Gadsden Boat Ramp at that time. For example, 22 km to the south at Neely Henry Dam (Population #6), we collected fewer red shiner (24%) than at a distance of 32 km south (Rainbow Landing, Population #7), where 50% of individuals collected were red shiner (Figs 1 and 2). A greater proportion of admixed individuals were also collected from Population #7 (Figs 1 and 2). We subsequently extended our sampling transect in 2011 another 56 km further downstream to include five sites around Logan Martin Lake (AL). We found that the downstream distributional limit of red shiner in 2011 fell within the reservoir; red shiner was only found at the most northern site in the reservoir (Stemley Bridge, Site #5). Sites further downstream did, however, harbor low frequencies of hybrids (Figs 1 and 2). For example, we recovered one putative F1 hybrid and one backcross at the southern terminus of the transect (Glover's Ferry, Population #1) just past Lower Logan Martin Dam. According to the southward cline models estimated for 2011 collections, the center of the phenotypic cline was located 90 km south of Brushy Branch (i.e., the northern terminus), and the cline exhibited a width of 57 km (Table 2, Fig. 4). The mtDNA haplotype model estimated a more southern cline center (117 km) and a much wider width of 144 km (Table 2, Fig. 4). Similarly, the multilocus genotype model estimated a cline center located 123 km from the northern terminus, and a width of 150 km (Table 2, Fig. 4).

## Discussion

Hybrid swarms that form following the introduction of a non‐native species can decrease biodiversity by overwhelming native taxa (Huxel 1999; Epifanio and Philipp 2000; Hall et al. 2006; Ward et al. 2012). The potential for loss of native biota through biological invasions involving hybridization has risen as human‐mediated introduction, and invasions of non‐native species have increased over time (Epifanio and Philipp 2000; Allendorf et al. 2001; Crispo et al. 2011). Although prior studies examining the *C. lutrensis x C. venusta* hybrid swarm in the Upper Coosa River basin (Walters et al. 2008; Ward et al. 2012) have characterized the formation of the swarm, as well as the spatial extent and factors contributing to hybridization (Blum et al. 2010; Ward and Blum 2012; Glotzbecker et al. 2015), little information has been available on the stability and evolution of the swarm over time. Empirical analysis of phenotypic and genetic clines across hybrid swarms over time is arguably a more reliable method for assessing stability, including movement and expansion (Blum 2002, 2008; Dasmahapatra et al. 2002; Buggs 2007). Here, we examined the spatio‐temporal dynamics of the *C. lutrensis x C. venusta* hybrid swarm to assess prior inferences of movement and expansion based on historical collection records. According to the findings of previous studies (Walters et al. 2008; Ward et al. 2012), we expected to see progressive northward movement and expansion. As expected, we found evidence of significant northward shifts in cline centers across a six‐year period and detected a modest increase in overall estimates of hybridization. Additionally, hybrids were detected farther north as years progressed. We also detected the presence of hybrids farther downstream from the historical introduction site than prior surveys have found. Notably, we did not find evidence of progressive expansion of the swarm. Rather, we detected a signature of contraction and expansion, suggesting that the stability and size of the swarm fluctuate over time, likely as a consequence of temporal shifts in extrinsic and intrinsic drivers of hybridization.

### Hybridization over time

Elevated fitness of hybrid genotypes and the rise of advantageous traits in admixed populations can hasten the erosion of species boundaries or genetic assimilation of parental entities (Arnold 1997; Barton 2001; Coyne and Orr 2004; Hall et al. 2006). Blum et al. (2010) showed that *C. lutrensis x C. venusta* hybrids exhibit comparable or higher measures of postzygotic fitness than offspring of parental species under laboratory conditions, suggesting that the proportion of hybrids could rise over time in the Upper Coosa River basin. We found that the majority of hybrids in the system were either later‐generation or backcrossed individuals (Fig. 2), confirming prior inferences that once formed, *C. lutrensis x C. venusta* hybrids persist (Walters et al. 2008; Blum et al. 2010; Ward et al. 2012). We also observed a 21% increase in the proportion of hybridization over the 6‐year study period, confirming prior predictions that hybrids would become more dominant over time (Walters et al. 2008; Blum et al. 2010). Additionally, the proportions of red shiner and hybrids at northward sites along the transect have increased across the sampling period (Figs 1 and 2, Figure S1). For example, in 2005 no evidence of hybridization was found at a distance of 332 km from the southern terminus (i.e., East Gadsden Boat Ramp) of the transect, whereas hybrid genotypes were detected at comparable distances in 2011. This suggests that the observed increase in hybridization is not limited to the rise of hybrids within a localized nucleus of sites (i.e., the center of the hybrid swarm), but rather further dispersion of red shiner and hybrids throughout the system. In agreement with theoretical predictions (Endler 1977; Huxel 1999; Hall et al. 2006), this finding suggests that increases in the frequency of successfully reproducing later‐generation hybrids are reshaping the boundaries and fueling the movement of the *C. lutrensis x C. venusta* hybrid swarm. Evidence of increasing hybridization contributing to rapid spread in the Coosa River system parallels findings of prior studies on biological invasions involving hybridization (Hovick and Whitney 2014) and patterns of longitudinal dispersal of nonmigratory fishes in river–stream networks (e.g., Waits et al. 2008; Lamphere and Blum 2012). For example, Childs et al. (1996) found that the frequencies of introduced alleles in populations of hybrid pupfish (*Cyprinodon pecosensis* x *C. variegatus*) increased over the course of a six‐year period in the Pecos River (Texas, USA). Similarly, studies examining populations of hybrid cordgrass (*Spartina alterniflora* x *S. foliosa*) in San Francisco Bay (California, USA) documented a rapid increase in hybrid genotypes, including pulsed increases over a 25‐year period (Ayres et al. 2008a,b; Strong and Ayres 2013), where more than a twofold increase in the percentage of hybrid area cover has been observed in local marshes in as little as one‐year time (Ayres et al. 2008a,b).

### The southern extent of the swarm

Prior studies of red shiner and *C. lutrensis x C. venusta* hybrids in the Upper Coosa River basin have not examined the potential for downstream spread from the point of introduction (Walters et al. 2008; Ward et al. 2012). Red shiners were first collected in Weiss Lake in 1974 and subsequently collected downstream in nearby Terrapin Creek, a tributary of the ‘Dead River’ arm of the Coosa River, in 1982 (Walters et al. 2008). Collection records indicate that red shiner or hybrids progressed upstream of Weiss Lake as early as 1992, when they were collected in Coahulla Creek (Walters et al. 2008). Collections in 1998 revealed an extensive hybrid swarm extending upstream from Weiss Lake to the confluence with the Conasauga River (Walters et al. 2008). Subsequent annual surveys (Walters et al. 2008; Ward et al. 2012) documented progressive spread of red shiner and *C. lutrensis x C. venusta* hybrids to areas of the upper Conasauga River that harbor the largest remaining population of federally threatened blue shiner (*Cyprinella caerulea)*. Our recovery of red shiner and hybrid genotypes at distances of >200 km downstream of Weiss Lake, and model estimates indicating that clinal transitions extend >100 km south of Weiss Lake (Table 2), indicates that red shiner and *C. lutrensis x C. venusta* hybrids pose a comparable threat to vulnerable congeners elsewhere in the system. Downstream spread in the Coosa River is thus a greater conservation concern than previously thought, particularly to remnant populations of blue shiner in tributaries that feed in to Weiss Lake or further downstream in to the mainstem Coosa River.

### Clinal coincidence, concordance, and stability over time

Traits under different selection regimes are expected to introgress across species boundaries at different rates (Harrison 1990; Mallet 2005; Yuri et al. 2009). For example, attributes under neutral or positive selection are expected to introgress more so than traits under divergent selection (Gay et al. 2009; Maroja et al. 2009; Ward et al. 2012). Accordingly, we found wider clines in multilocus genotype admixture profiles and mtDNA haplotypes compared to the clines describing variation at phenotypic traits that reflect functional differences among hybridizing taxa (Ward and Blum 2012). Similar to prior findings (Ward et al. 2012), our results indicate that microsatellite alleles and mtDNA haplotypes are introgressing more extensively than phenotypic traits (Fig. 3, Figure S1). We also found a close correspondence between multilocus genotype and mtDNA haplotype (Figs 2 and 3). Nonetheless, comparison with theoretical values for neutral traits suggests that constraints are limiting the diffusion of alleles, haplotypes, and morphological attributes (Endler 1977). As *C. lutrensis* can produce as many as two generations per year (Farringer et al. 1979), and given a maximum estimated dispersal rate of ~31 km per year (Walters et al. 2008; and herein), the approximate width of neutral clines could be as broad as 700 km (Endler 1977; Ward et al. 2012). Thus, clines have remained narrower than expected under neutral diffusion, suggesting that there are factors structuring introgression across the hybrid swarm (Endler 1977; Gay et al. 2007; Ward et al. 2012).

Correspondence between phenotype and mtDNA haplotype clines found in prior work (Ward et al. 2012) suggested that maternal contributions could be constraining introgression and that *C. lutrensis* traits are selectively favored. Overall, the cline estimates reported here provide qualitatively similarly, but more conservative estimates of movement and instability compared to the values presented in Ward et al. (2012). We attribute these differences to the use of different modeling approaches to estimate cline attributes. Following the approaches taken in, Ward et al. (2012) would have yielded higher estimates of the extent and rate of movement as well as the extent of expansion over the study period. Nonetheless, evidence that the phenotypic cline has remained narrower than either of the mtDNA and multilocus nuclear clines provides further support for the inference that selection is structuring phenotypic introgression across the hybrid swarm (Ward et al. 2012). Evidence that the phenotypic cline has been advancing ahead of the mtDNA and multilocus nuclear clines also indicates that the red shiner phenotype confers selective advantages, likely reflecting short generation times, an aggressive disposition, and higher fecundity (DeVivo 1995; Bensch et al. 1999; Fuller et al. 1999; Balloux et al. 2000; Burkhead and Huge 2002; Rees et al. 2003; Vallender et al. 2007; Brelsford and Irwin 2009; Blum et al. 2010; Ward et al. 2012). This inference is supported by a meta‐analysis of biological invasions involving hybridization (Hovick and Whitney 2014) suggesting that hybrids often exhibit phenotypes that confer higher fecundity, larger body size, and equal survival when compared to parental phenotypes. However, we also found that the hybrid swarm did not progressively expand over time, which indicates that other factors are influencing introgression. Evidence of contraction and expansion suggests that the swarm may fluctuate in response to temporal shifts in extrinsic drivers of hybridization such as impairment of water quality characteristics that influence reproductive isolation or that result in conditions that favor hybrids (Guo 2014). Prior work indicates that shifts in either turbidity or chemical contamination could influence the amount and distribution of hybridization between native *C. venusta* and invasive *C. lutrensis* over time (Blum et al. 2010; Ward and Blum 2012; Glotzbecker et al. 2015).

Accurate estimates of tempo and rates are required for determining the factor(s) promoting movement (Barton and Hewitt 1985, 1989; Blum 2002; Buggs 2007) such as selective advantage of one phenotype over others in a given environment (Goodman et al. 1999), dominance drive (Blum 2002, 2008), asymmetric hybridization (Bronson et al. 2003; Buggs and Pannell 2006), hybrid fitness (Klingenberg et al. 2000), anthropogenic environmental disturbance (Blum 2002), and climate change (Britch et al. 2001; Taylor et al. 2014). Annual surveys of the Conasauga River have indicated that red shiner and *C. lutrensis x C. venusta* hybrids can disperse at rates up to 31 km per year (Walters et al. 2008). We recovered a comparable maximum rate of 32 km per year according to the movement of the phenotypic cline between 2005 and 2008, although cline models indicate that upstream movement of the *C. lutrensis x C. venusta* hybrid swarm has generally proceeded at a slower pace than suggested by collection records. For instance, between 2005 and 2011, the center of the phenotypic cline advanced approximately 133 km upstream at an average rate of 22 km per year. The centers of the mtDNA and multilocus genotypic clines advanced at an even slower pace of approximately 8–12 km per year. Comparison of cline centers over time also suggests that the rate of upstream movement has been declining. The center of the phenotypic cline proceeded upstream at a rate of 32 km per year between 2005 and 2008 compared to a rate of 12 km per year between 2008 and 2011. The centers of the mtDNA and genotypic clines moved at a rate of 15–21 km per year between 2005 and 2008, and effectively remained stable between 2008 and 2011.

It is possible that the upstream advance of the hybrid swarm is slowing because red shiner and hybrids are encountering unfavorable ecological conditions. Red shiner tends to prefer warmer, low‐elevation habitats with sand or silt substrate (Matthews and Hill 1979). The Conasauga River transitions to cooler and higher elevation conditions at a distance of approximately 352 km from East Gadsden Boat Ramp. Past the Georgia‐Tennessee state line, the substrate of the Conasauga River is also largely composed of large boulders and sedimentary limestone. Although further monitoring and experimental tests are warranted to test this inference, it is likely that habitat transitions could eventually halt further upstream movement of the *C. lutrensis x C. venusta* swarm (Buggs 2007; Blum 2008), and possibly prevent further overlap with threatened blue shiner in the Conasauga River.

### Conservation and management implications

While anthropogenic disturbance has been consistently linked to the replacement of species pairs by hybrid swarms, the mechanisms leading from disruptive change to species collapse, and the long‐term effects of hybridization on species pairs remain poorly understood (Gilman and Behm 2011). The invasive *C. lutrensis x C. venusta* hybrid swarm in the Upper Coosa River basin affords exceptional opportunities to understand the formation and evolution of hybrid swarms over time. Consistent with theoretical predictions that invasions driven by evolutionary processes are likely to exhibit stochastic rates of spread (Phillips 2015), our findings indicate that the *C. lutrensis x C. venusta* hybrid swarm is continuing to advance, but that the movement of phenotypic, mtDNA, and genotypic clines have been progressing at different rates over time. We also found evidence that, despite directional movement, the extent of the hybrid swarm has fluctuated over time and that ecological constraints may impede further upstream progress. For the first time, we also documented the downstream extent of red shiner and hybrids ≥200 km south of the original introduction site at Lake Weiss. These findings suggest that, even if conditions of stochasticity prevail, should further upstream progress be constrained, invasive red shiner and hybrids are likely to advance further downstream in the Coosa River, which could threaten two of the four remaining populations of federally threatened blue shiner. Further, downstream spread could also present greater opportunity for hybridization with other native species of *Cyprinella* found in the Upper Coosa River system. Although to date there is no documented evidence of invasive red shiner hybridizing with either the tricolor shiner (*C. trichroistia*) or the Alabama shiner (*C. callistia*), non‐native red shiner has a remarkable propensity to hybridize with native congeners (Walters et al. 2008). Thus, additional investigation of hybridization downstream of Weiss Lake is warranted, and monitoring at both the upstream and downstream leading edges of the hybrid swarm could offer further guidance for prioritizing efforts to protect native *Cyprinella* and identify factors that could halt or mitigate future spread to areas of conservation concern in the Coosa River basin.

## Data archiving

Data available from the Dryad Digital Repository: http://dx.doi.org/10.5061/dryad.s6k78

## Supporting information


**Figure S1** (A–C) Clinal changes in the frequencies of *Cyprinella* phenotype, mtDNA haplotype, and microsatellite multilocus genotype. (A) 2005 upstream cline models, (B) 2008 upstream cline models, (C) 2011 upstream cline models. East Gadsden Boat Ramp served as the southern terminus of the transect over which the clines were estimated.
