# Supplementary figures and images for: Rapid movement and instability of an invasive hybrid swarm
Source: Evol Appl. 2016 Apr 27;9(6):741–55. doi: 10.1111/eva.12371 (PMC4908461; doi:10.1111/eva.12371)

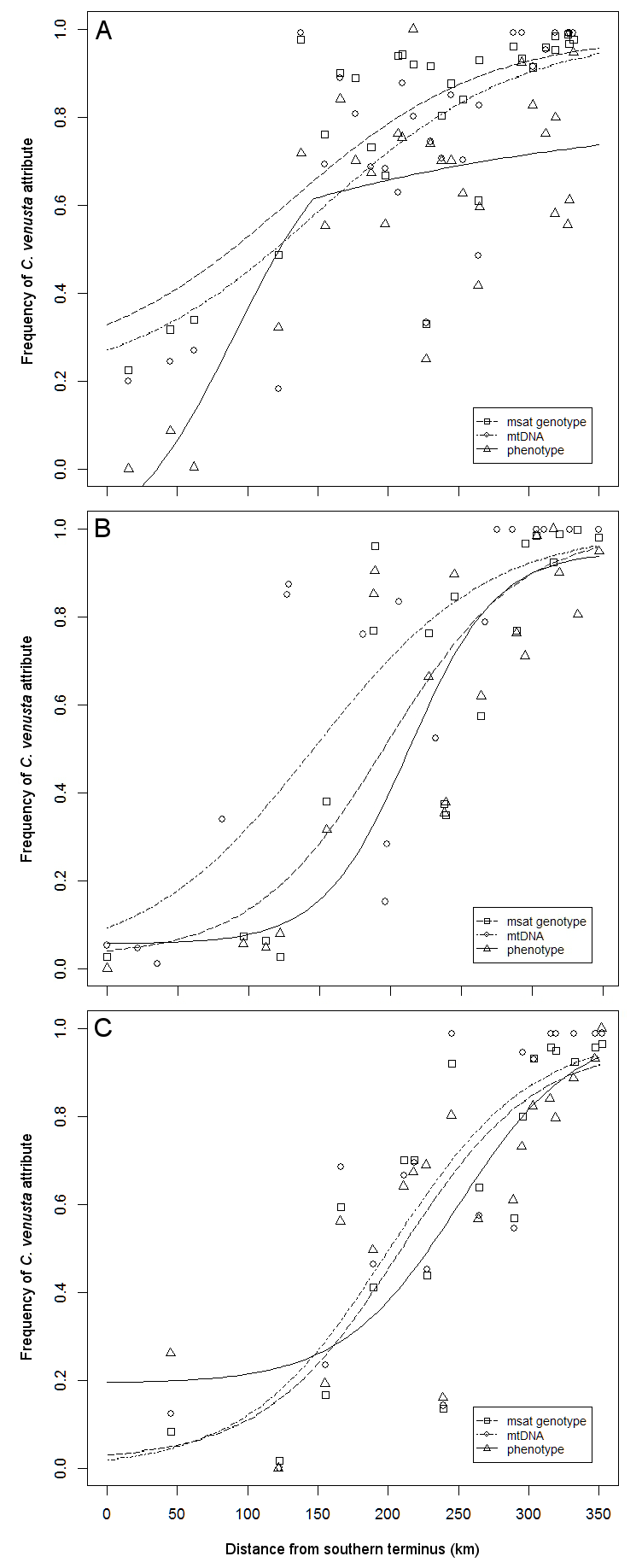

Supplement: Supplementary file 1 — Figure S1 (A–C) Clinal changes in the frequencies of Cyprinella phenotype, mtDNA haplotype, and microsatellite multilocus genotype. (A) 2005 upstream cline models, (B) 2008 upstream cline models, (C) 2011 upstream cline models. East Gadsden Boat Ramp served as the southern terminus of the transect over which the clines were estimated. [file EVA-9-741-s001.png]
